# Identification and characterization of tomato gibberellin 2-oxidases (GA2oxs) and effects of fruit-specific *SlGA2ox1* overexpression on fruit and seed growth and development

**DOI:** 10.1038/hortres.2016.59

**Published:** 2016-12-07

**Authors:** Shen Chen, Xiaojing Wang, Liying Zhang, Shanshan Lin, Decai Liu, Quanzhi Wang, Shanya Cai, Rania El-Tanbouly, Lijun Gan, Han Wu, Yi Li

**Affiliations:** 1 State Key Laboratory of Crop Genetics and Germplasm Enhancement, College of Horticulture, Nanjing Agricultural University, Nanjing 210095, China; 2 College of Life Sciences, Nanjing Agricultural University, Nanjing 210095, China; 3 Jiangsu Polytechnic College of Agriculture and Forestry, Zhenjiang 212400, China; 4 Jiangsu Engineering and Technology Center for Modern Horticulture, Zhenjiang 212400, China; 5 Department of Plant Science and Landscape Architecture, University of Connecticut, Storrs, CT 06269, USA

## Abstract

Gibberellins (GAs) play a crucial role in growth and development of the tomato fruit. Previously published studies focusing on the effect of GAs on tomato fruits used chemical treatments, constitutive overexpression or silencing of GA biosynthetic and catabolic genes globally throughout the plant. Fruit-specific overexpression of GA catabolic enzyme genes *GA2-oxidases* (*GA2oxs*), however, may provide an alternative method to study the role of endogenous GAs on the fruit development. In this study, we have identified 11 SlGA2ox proteins in tomato that are classified into three subgroups. Motif analysis and multiple sequence alignments have demonstrated that all SlGA2oxs, except SlGA2ox10, have similar motif compositions and high-sequence conservation. Quantitative reverse transcription-PCR analysis has showed that *SlGA2oxs* exhibit differential expression patterns in tomato fruits at different developmental stages. When the fruit-specific promoter TFM7 was used to control the expression of *SlGA2ox1*, we observed no changes in growth and development of vegetative organs. However, fruit weight, seed number and germination rate were significantly affected. We also treated tomato fruits with GA biosynthesis inhibitor and observed phenotypes similar to those of the transgenic fruits. Furthermore, we have demonstrated that expression of cell expansion and GA responsive genes were downregulated in transgenic tomato fruits, supporting that overexpression of the *SlGA2ox1* leads to reduction in endogenous GAs. This study provides additional evidence that endogenous GAs and the *SlGA2ox1* gene play an important role in controlling on fruit weight, seed development and germination in tomato plant.

## Introduction

Tomato (*Solanum lycopersicum* L.) is an important vegetable crop worldwide, and has been used as a model plant for basic research on fruit growth and development.^1^ Plant hormones participate in the growth and development of tomato organs, including fruits and seeds.^2–4^ Gibberellins (GAs) are important hormones that control fruit set and growth, and seed development in tomato.^5–7^

Among the several hundred plant GAs, only a few (such as GA_1_, GA_3_, GA_4_ and GA_7_) are biologically active in higher plants. GA_1_ and GA_4_ are the major bioactive GAs with relatively high abundance in various plant species, while GA_3_ and GA_7_ are less abundant.^8^ The intracellular concentrations of active GAs are regulated by the balance between rates of biosynthesis and deactivation. The GA biosynthesis pathway is catalyzed by a series of metabolic enzymes, such as *ent*-copalyl diphosphate synthase (CPS), *ent*-kaurene synthase (KS), *ent-*kaurene oxidase (KO), *ent-*kaurenoic acid oxidase (KAO), GA20-oxidase (GA20ox) and GA3-oxidase (GA3ox). GA20ox and GA3ox enzymes are encoded by small gene families that catalyze the rate-limiting steps in GA biosynthesis.^8–10^ In the GA deactivation pathway, bioactive GAs or their immediate precursors are converted into inactive forms by GA2 oxidases (GA2oxs), including C19-GA2oxs and C20-GA2oxs. The substrates of C19-GA2oxs, C19-GAs (GA_1_ and GA_4_) and their precursors (GA_20_ and GA_9_) can be converted to inactive GAs (GA_8_, GA_34_, GA_29_ and GA_51_). In contrast, the substrates of C20-GA2oxs, which are C20-GAs (GA_12_ and GA_53_), can be hydroxylated to the inactive forms GA_110_ and GA_97_, respectively.^8–10^

Several *GA2oxs* have been identified and are functionally characterized in *Arabidopsis* and rice.^11–21^ Manipulating the expression of *GA2ox* genes enables regulation of the levels of endogenous active GAs in some plant species. For example, overexpression of *GA2ox* genes in plants causes deficiency in endogenous GAs, leading to dwarf plants.^17–22^ Also, overexpression of *AtGA2ox1* in bahiagrass improves turf quality under field conditions.^23^ Furthermore, overexpression of *AtGA2ox8* in canola results in semi-dwarf plants with a high seed oil content.^24^

In tomato fruits, GAs are important hormones that accumulate during fruit cell division and cell expansion at early developmental stages.^2^ Application of exogenous GA_3_ to unpollinated ovaries of tomato increases the fruit set, but the fruits are parthenocarpic with reduced sizes of locular tissues and fruits compared with pollinated fruits.^22^ Conversely, exogenous treatment of pollinated ovaries with GA biosynthesis inhibitors decreases the fruit set, and the fruits are smaller than untreated pollinated fruits.^6,25–27^ In addition, modifying the expression of the genes that regulate GA biosynthesis and deactivation can influence the endogenous GA levels and vegetative growth of tomato plants. Overexpression of the citrus gene *CcGA20ox1* in tomato plants increases GA_4_ levels, and the transgenic plants are taller with non-serrated leaves. The transgenic plants produce parthenocarpic fruits and the fruit yield is increased.^28^ In contrast, tomato plants in which *SlGA20ox1* was silenced have deformed leaves and reduced fertility.^29^ Xiao *et al.*^30^ reported that suppression of *SlGA20ox1* or *SlGA20ox2* in tomato results in shorter stems and smaller but dark green leaves.^30^ It has been shown that downregulating the expression of five C19 *GA2ox* genes in tomato results in a significant increase in active GA_4_ content, and the transgenic tomato plants have parthenocarpic fruits, with reduced lateral branching.^31^

In this study, we identified GA2ox proteins in tomato, constructed a phylogenetic tree, and performed protein motif organization analysis and multiple sequence alignments (MSAs). We also examined the expression patterns of *SlGA2ox* genes using quantitative reverse transcription-PCR (qRT-PCR). Finally, we used a fruit-specific promoter, TFM7, to control the expression of *SlGA2ox1* in the tomato cultivar ‘Micro-Tom’. The results provide valuable information on the roles of GAs and *SlGA2ox1* gene in the growth and development of tomato fruits and seeds.

## Materials and methods

### Plant material and hormone treatments

Tomato (*Solanum lycopersicum*) ‘Micro-Tom’ was used in this study. Tomato plants were grown in a climate-controlled greenhouse at 25 °C for 14 h during the day, followed by 20 °C for 10 h during the night at 60% relative humidity. GA_3_ (10^−4^ M) and paclobutrazol (PAC) were applied to tomato plant roots in nutrient solution after pollination. Plants were treated once per day for 10 days. At least eight fruits were analyzed per treatment. The fruit weight of fully ripened fruits was measured.

### Identification of GA2ox proteins in tomato

GA2ox protein sequences of *Arabidopsis* and rice that were identified and functionally characterized in previous studies were downloaded from the National Center for Biotechnology Information (NCBI) (http://www.ncbi.nlm.nih.gov). The Pfam database v28.0^32^ was used to analyze the conserved domains of these GA2ox proteins. The *S. lycopersicum* genome sequence and corresponding annotations were downloaded from the US Department of Energy Joint Genome Institute (DOE JGI) website (http://genome.jgi.doe.gov). The protein entries matching the conserved domains 2OG-FeII_Oxy (PF00847) and DIOX_N (PF14226) were identified using HMMER^33^, with an E-value cut-off of 10^−4^.

### Phylogenetic trees, motif recognition and MSAs of GA2ox proteins

The amino-acid sequences of GA2oxs from various plant species were downloaded from the NCBI database, and aligned using ClustalW. A mid-point phylogenetic analysis was performed based on the maximum-likelihood method using PHYML version 3.0 under the JTT evolution model. The reliability of the obtained trees was tested by bootstrapping with 100 replicates. Phylogenetic trees were visualized with FIGTREE (http://tree.bio.ed.ac.uk/software/figtree/). Motif analysis was performed using the MEME software (http://meme-suite.org/), with the default parameters.^34^ Multiple-sequence alignments were performed with a gap open penalty of 10 and gap extension penalty of 0.2 using DNAMAN 6.0 and ClustalW 2.0.^35^

### Construction of plasmids and plant transformation

The fragment of TFM7 promoter was amplified using primers (forward primer: 5′- ACGGCCAGTGCCAAGCTTACTGACCCCAATCAAGC-3′ and reverse primer: 5′- ACGATCTCTAGAGTCGACTGGTATGGAGAGAGGGA-3′) from DNA of ‘Micro-Tom’, and inserted into the *Hin*dIII and *Sal*I sites of binary vector pCAMBIA2300, then the NOS terminator was inserted into the *Xba*I and *Bam*HI sites. At last, the complete coding sequence of *SlGA2ox1* was amplified using primers (forward primer: 5′- TCCCTCTCTCCATACCAGTCGACATGGTTTCTGAAAAAATTCAAG-3′ and reverse primer: 5′- CAAATGTTTGAACGATCTCTAGATTAAGACGTTTGATTATTAATAG-3′) from cDNA of ‘Micro-Tom’ and cloned into the *Sal*I and *Xba*I sites. Transgenic ‘Micro-Tom’ tomato plants were generated by *Agrobacterium tumefaciens*-mediated (strain EHA105) transformation using methods described previously.^36^

### Germination assays

Germination experiments were conducted in a growth chamber. Seeds of wild-type and transgenic tomato plants were incubated in sterile water at 28 °C in the dark. The germination percentage was calculated by counting seeds with the first sign of radicle tip appearance after 48, 60 and 72 h of incubation. PAC (10^−5^ M) and GA_3_ (10^−5^ M) were applied to 40 seeds per sample, with three replicates per treatment.

### RNA isolation and real-time quantitative PCR assays

Total RNA was extracted using an RNeasy Plant Mini Kit (Takara, Dalian, Liaoning, China). Extracted RNA was treated with RNase-Free DNAse according to the manufacturer’s instructions. Total DNA-free RNA was used as a template for cDNA synthesis, using the Primerscript RT Reagent Kit (Takara, Dalian, Liaoning, China) with gDNA Erase (Takara, Dalian, Liaoning, China). For real-time quantitative PCR, SYBR Premix Ex Tag was used on a Bio-Rad CFX96 (Bio-Rad, Shanghai, China) instrument. PCR reactions were performed in a 96-well iCycler. The melting temperature of the products was determined to verify the specificity of the amplified fragments. The sequences of primers used for real-time quantitative PCR are listed in Supplementary Table 1. Results were analyzed by the ΔΔCT method using *SlActin* as the control locus. All real-time quantitative PCR data points were the average of three biological replicates, and three technical replicates.

## Results

### Genome-wide identification and analysis of GA2ox proteins in tomato

Several *GA2ox* genes of *Arabidopsis* and rice have been isolated and functionally analyzed.^11–21^ Using the Pfam database, two conserved domains [2OG-FeII_Oxy (PF00847) and DIOX_N (PF14226)] were detected in all GA2ox proteins. The two conserved domains were used as queries for HMMER searches of the tomato genome. After removing redundant proteins, a total of 131 putative candidate proteins was identified. A phylogenetic tree was constructed using the 131 identified proteins from tomato and the previously identified GA2ox proteins of *Arabidopsis* and rice. Eleven proteins of tomato were assigned to the GA2ox family (data not shown). Five (SlGA2ox1-SlGA2ox5) have been reported previously,^6^ and six GA2oxs, designated here as SlGA2ox6 to SlGA2ox11, were newly identified in this study.

**Figure 1:**
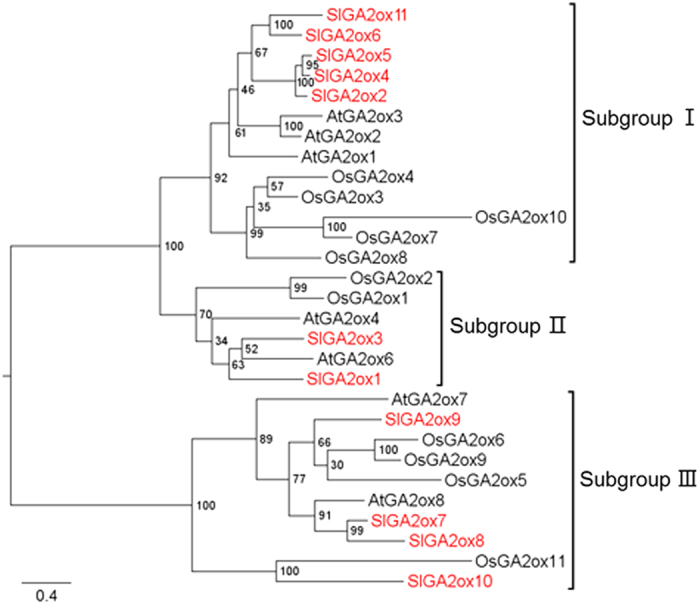
Phylogenetic tree of GA2-oxidase protein sequences from *Arabidopsis*, rice and tomato. GA2oxs were divided into three subgroups: subgroup I, subgroup II and subgroup III. GA2ox proteins from tomato were marked in red color. The amino-acid sequences of GA2ox proteins were aligned using ClustaIW and a phylogenetic tree was constructed by PhyML software using the ML method with 100 bootstraps.

We performed a BLASTP search in the NCBI database using tomato GA2ox amino-acid sequences in the Pfam database. The most reliable amino-acid sequences of GA2oxs were downloaded, and a second phylogenetic tree was constructed (Figure 1). The tree topology revealed that GA2ox proteins could be divided into subgroups I, II and III. Subgroups I and II belong to C19 GA2oxs, and subgroup III belongs to C20 GA2oxs. Proteins in subgroups I and II are more closely related to each other than to those in subgroup III. In tomato, five GA2ox proteins (SlGA2ox2, SlGA2ox4, SlGA2ox5, SlGA2ox6 and SlGA2ox11) were classified as belonging to subgroup I as they had the highest homology with AtGA2ox1, AtGA2ox2 and AtGA2ox3. These proteins clustered together to form a monophyletic group, which suggests they emerged through lineage-specific expansion events in tomato. In addition, SlGA2ox1 and SlGA2ox3 were assigned to subgroup II and were more closely related to AtGA2ox6. Furthermore, four GA2ox proteins in tomato (SlGA2ox7 to SlGA2ox10) belonged to subgroup III, of which SlGA2ox7 and SlGA2ox8 were orthologous to AtGA2ox8. Also SlGA2ox9 was orthologous to OsGA2ox5, OsGA2ox6 and OsGA2ox9. SlGA2ox10 and OsGA2ox11 were orthologous in the same branch of the phylogenetic tree.

On the basis of the above classification, the 11 tomato GA2ox proteins were subjected to further analysis. More detailed information was collected, including the protein ID in NCBI, chromosomal distribution, sequence length, isoelectric point (pI) and protein molecular mass (Supplementary Table S2). The *GA2ox* genes were unevenly distributed on the tomato chromosomes. *SlGA2ox2*, *SlGA2ox4* and *SlGA2ox5* were on chromosome 7, *SlGA2ox6* and *SlGA2ox7* were on chromosome 2, *SlGA2ox3*, *SlGA2ox9*, *SlGA2ox1*, *SlGA2ox10*, *SlGA2ox11* and *SlGA2ox8* were on chromosomes 1, 4, 5, 6, 8 and 10, respectively. The identified GA2ox proteins ranged from 322 to 380 amino acids in length, had pI values of 5.15 to 9.17, and protein molecular mass of 36.05–44.12 kDa. In addition, the similarity percentage between the different SlGA2ox proteins ranged from 25.00 to 85.94% (Supplementary Table S3).

### Motif analysis and MSAs of GA2ox proteins

**Figure 2:**
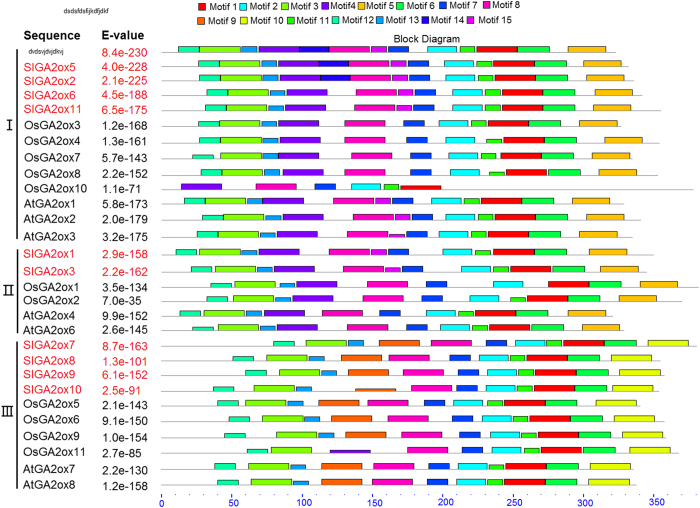
Conserved motif analysis of GA2ox proteins from *Arabidopsis*, rice and tomato. Motifs were identified using MEME software. Fifteen motifs were identified and indicated by boxed of different color, with the distributions of each motif corresponding to their position. The names and combined E values of genes are shown the left side. Gray lines represent non-conserved sequences. The names of GA2ox proteins from tomato are marked in red color.

The conserved motifs of GA2ox proteins were extracted using the MEME software. All of the GA2ox proteins from *Arabidopsis*, rice and tomato had a similar motif composition (Figure 2; Supplementary Figure S1). A total of 15 motifs (motifs 1–15) were identified, including nine highly conserved motifs (motifs 1, 2, 3, 6, 7, 8, 11, 12 and 13) detected in all GA2ox subgroups, with the exception of OsGA2ox10 protein. In addition, results suggest the existence of subgroup-specific motifs, of which motifs 4 and 5 were specific to subgroups I and II, and motifs 9 and 10 were specific to subgroup III, with the exception of OsGA2ox11. Because subgroups I and II belonged to C19 GA2ox and subgroup III belonged to C20 GA2ox, the subgroup-specific motifs of the C19 and C20 GA2ox families may be involved in the deactivation of different GAs. All tomato GA2ox proteins had all of the motifs present in GA2oxs of *Arabidopsis* and rice; however, the sequence of SlGA2ox10 at the motif 9 position was less conserved than the other proteins in the same group.

MSAs was performed to analyze the difference of conserved amino acid sequences of the GA2ox proteins of *Arabidopsis*, rice and tomato (data not shown). There was no significant variation between subgroups I and II, however, a significant sequence variation between C19 GA2oxs and C20 GA2oxs was identified, particularly in motifs 4 and 9 and motifs 5 and 10, respectively. All of the GA2ox proteins contained several conserved sites, including two putative 2-oxoglutarate-binding sites and three iron-binding sites. However, OsGA2ox10 lacked one 2-oxoglutarate-binding site and one iron-binding site, its amino-acid sequence varied significantly compared to the other GA2ox proteins, although it grouped with the GA2ox family in the phylogenetic tree.

To predict whether putative GA2oxs from tomato have GA2 oxidase activity, OsGA2ox2, OsGA2ox4, OsGA2ox7, OsGA2ox8, OsGA2ox10 and OsGA2ox11 were removed as their functions have not been identified, and replaced with other known GA2ox proteins from various plant species (Supplementary Figure S2). MSAs showed that SlGA2ox1, SlGA2ox2, SlGA2ox3, SlGA2ox4, SlGA2ox5, SlGA2ox6 and SlGA2ox11 have sequences similar to the other C19 GA2ox proteins, including C19 GA2ox-specific motifs 4 and 5 (Supplementary Figure S2A). In addition, motifs 9 and 10 contained three unique amino-acid sequences that were highly conserved among C20 GA2ox proteins. SlGA2ox7 and SlGA2ox8 shared all of the three unique sites in the subgroup-specific motifs. However, the predicted SlGA2ox9 varied in one amino acid at the first and second sites of unique sequence, while the third site was similar to those of other aligned proteins. Moreover, SlGA2ox10 exhibited one (position 126), four (position 154, 156, 158 and 160) and one (position 338) amino-acid variations at the first, second and third sites of the unique sequence, respectively, and the motif 9 position in the SlGA2ox10 sequence was significantly different compared to other GA2ox proteins (Supplementary Figure S2B). This suggests that SlGA2ox10 may not be able to deactivate C20 GAs.

### Expression patterns of *GA2ox* genes in tomato

**Figure 3:**
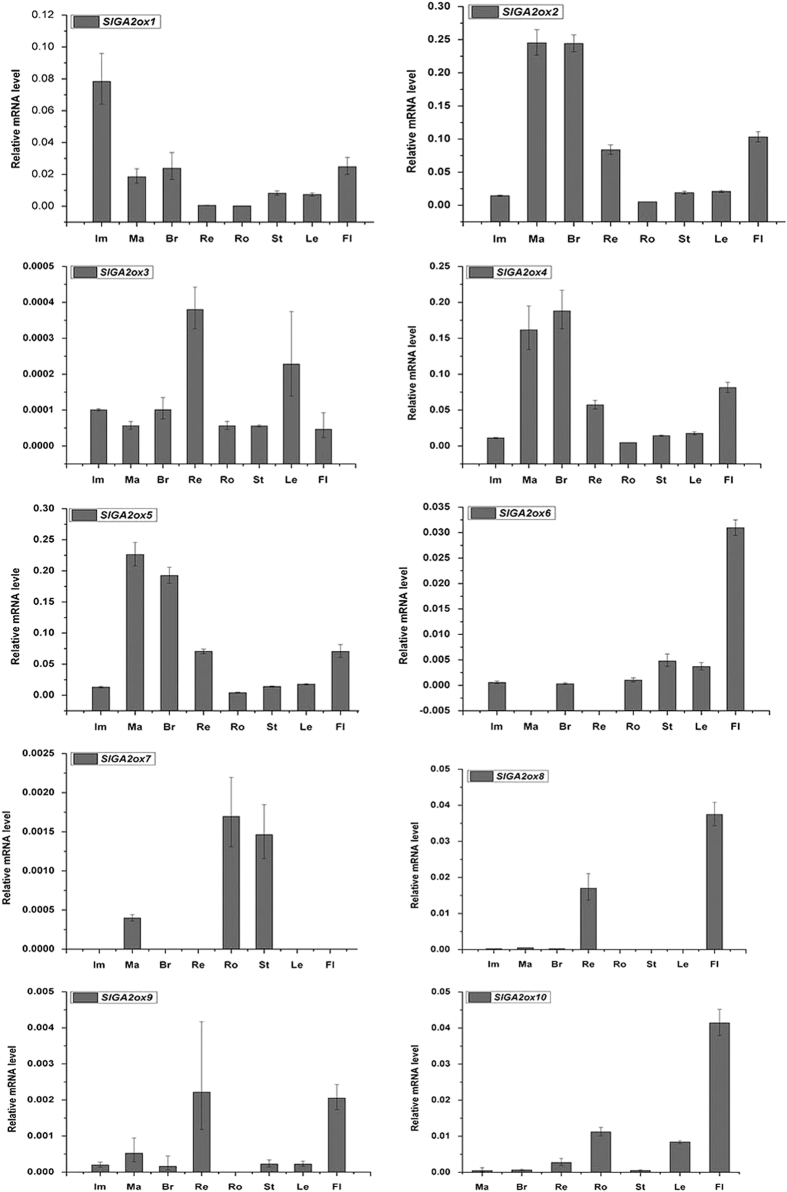
Expression patterns of GA2ox genes in tomato. Bars represent the relative abundance of *GA2ox* genes among the developmental stages of fruits and other organs. The *SlActin* gene was used as an internal control for each tissue and values are means of three biological replicates. Br, breaker fruits; Fl, flowers; Im, immature green fruits; Le, leaves; Ma, mature green fruits; Re, red ripening fruits; Ro, roots; St, stems.

qRT-PCR analysis was used to characterize the transcription profiles of *SlGA2ox* genes. The expression patterns of all of the *SlGA2ox* genes differed among the developmental stages of fruits and other organs (Figure 3). *GA2ox1* was highly expressed in fruits at immature green stage, but lowly expressed in fruits at red ripening stage and other vegetative organs, such as roots, stems and leaves. *SlGA2ox2* expression was high in flowers and fruits at mature green, breaker and red ripening stages, but low in immature green fruits, roots, stems and leaves. The expression of *SlGA2ox3* was high in red ripening fruits and leaves. *SlGA2ox4* and *SlGA2ox5* had similar expression patterns, they were high in fruits at mature green stage and post-breaker stages, but low in fruits at immature green stage and other vegetative organs. In addition, *SlGA2ox6* had the highest expression in flowers, but relatively low or no expression in developing fruits and other vegetative organs. *SlGA2ox7* mainly expressed in mature green fruits, roots and stems*, SlGA2ox8* mainly expressed in red ripening fruits and flowers. Furthermore, *SlGA2ox9* expression was relatively high in flowers and red ripening fruits, and low in fruits at other developmental stages and other vegetative organs. *SlGA2ox10* expression was high in flowers and low in other organs. *SlGA2ox11* expression was negligible at all developmental stages of fruits and other organs (data not shown).

### Effects of fruit-specific overexpression of *SlGA2ox1* on fruit growth and development

**Figure 4:**
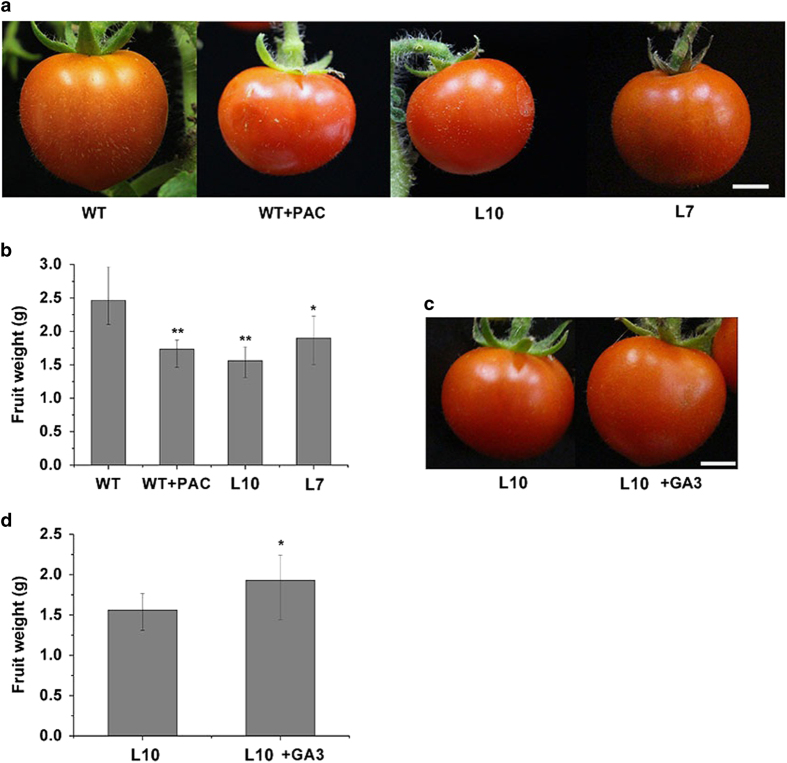
Effects of fruit-specific overexpression of *SlGA2ox1* on the fruit weight of transgenic tomato plants. (**a**) Phenotype of PAC-treated and Tfm7:*SlGA2ox1* transgenic tomato fruits. (**b**) Single-fruit weight of PAC-treated and transgenic tomato plants. (**c**) Phenotype of transgenic fruits treated with 10^−5^ M GA_3_. (**d**) Single-fruit weight of transgenic plants treated with 10^−5^ M GA_3_. Scale bars, 5 mm. *Significant difference by *t*-test, *P<0.05, **P<0.01. Data are means±s.d. (*n*=3).

Serrani *et al.*^6^ have reported that SlGA2ox1 can metabolize active GA_1_ and GA_4_ to inactive GA_8_ and GA_34_ through enzyme assay with recombinant protein,^6^ to determine whether the overexpression of *SlGA2ox1* in tomato fruits causes GA-deficient phenotypes, the fruit-specific promoter TFM7-SlGA2ox1 cDNA fusion construct was transformed into the tomato cultivar ‘Micro-Tom’. Because the TFM7 promoter is mainly active in fruits at early developmental stages,^37,38^ the growth and development of vegetative organs of transgenic plants were not affected (Supplementary Figure S3A). The expression of *SlGA2ox1* was evaluated in transgenic tomato fruits at 15 DAP. *SlGA2ox1* expression in transgenic tomato lines L10 and L7 was higher than in wild-type fruits (Supplementary Figure S3B). The single-fruit weights of transgenic lines L10 and L7 were 1.66±0.32 and 1.90±0.54 g, respectively, lower than the 2.44±0.72 g of wild-type fruits. Treatment with GA biosynthesis inhibitor (PAC) to wild-type fruits reduced the fruit weight to 1.74±0.33 g (Figures 4a and b). The reduced fruit weights of transgenic tomato could be restored by exogenous application of GA_3_ (Figures 4d and e), suggesting that the reduction in fruit weights observed in the transgenic tomato plants are due to reduction in active GA levels in fruits.

### Effects of fruit-specific overexpression of *SlGA2ox1* on seed development and germination in tomato

**Figure 5:**
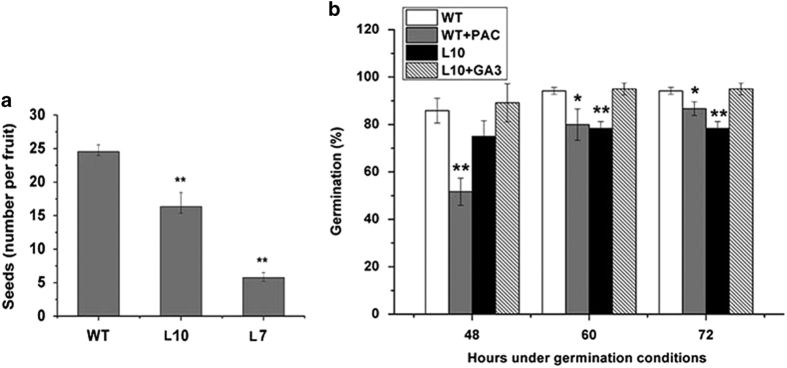
Effects of fruit-specific overexpression of *SlGA2ox1* on seed number and germination in transgenic tomato plants. (**a**) The average seed number of a single fruit of wild-type and transgenic plants. (**b**) The seed germination percentage of wild-type, PAC-treated, transgenic tomato and transgenic tomato seeds treated with 10^−5^ M GA_3_. *Significant difference compared with the wild type by *t*-test, *P<0.05, **P<0.01. Data are means±s.d. (*n*=3).

To determine the effect of *SlGA2ox1* overexpression in tomato fruits on seed development and germination, the seed number per fruit and germination rate were calculated. The seed number per fruit of transgenic lines L10 and L7 was 16.64±2.85 and 5.75±1.36, respectively, while that of wild-type fruit was 24.55±1.21 (Figure 4c). We observed that 85.83±5.20% of wild-type seeds germinated after 48 h, compared with 75.00±6.61% of transgenic seeds. After 60 h, 94.16±1.44% of wild-type seeds germinated, compared with 78.33±2.89% of transgenic seeds. Similarly, PAC reduced the seed germination rate of wild-type seeds (Figure 5). Furthermore, the germination rate of transgenic seeds was restored to 95.00±2.50% by exogenous application of GA_3_. These results suggest that fruit-specific overexpression of *SlGA2ox1* in tomato plants reduces the endogenous active GA concentrations in fruits, and that GAs are important regulators of tomato seed development and germination.

### Effects of fruit-specific overexpression of *SlGA2ox1* on the transcript levels of GA-regulated and cell expansion-related genes

**Figure 6:**
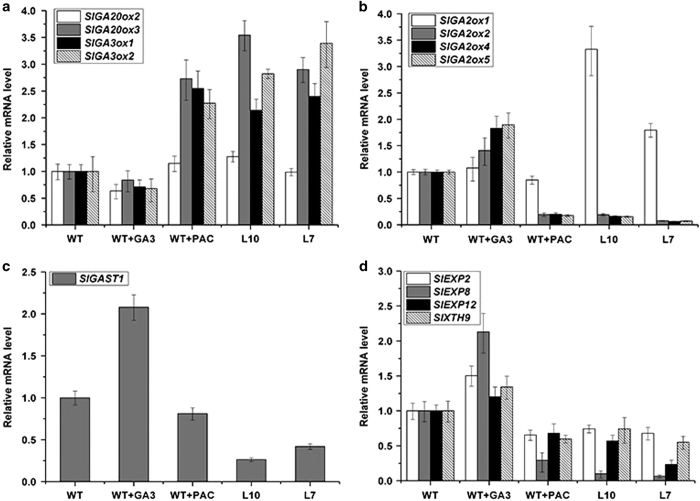
Transcript levels of GA- and cell expansion-related genes in tomato fruits treated with GA_3_ and PAC, and transgenic tomato fruits. (**a**) GA biosynthesis genes, (**b**) GA deactivation genes, (**c**) GA-responsive gene and (**d**) cell expansion-related genes. Data are means±s.d. (*n*=3).

The transcript levels of GA- and cell expansion-related genes in transgenic and wild-type fruits treated with GA_3_ and PAC, were determined using 15 DAP fruit tissues. The expression levels of GA biosynthesis-related genes (*SlGA20ox2*, *SlGA20ox3*, *SlGA3ox1* and *SlGA3ox2*) were reduced in GA_3_-treated fruits, and increased in PAC-treated and transgenic fruits comparing to wild-type fruits (Figure 6a). In contrast, the transcript levels of GA deactivation-related genes, *SlGA2ox2*, *SlGA2ox4* and *SlGA2ox5,* were increased in GA_3_-treated fruits, and decreased in PAC-treated and transgenic fruits compared to untreated wild-type fruits (Figure 6b). These results indicate that *SlGA2ox1* is responsible for GA deactivation and its overexpression influences the expression of GA-related genes.

*Gibberellins-stimulated transcripts1 (SlGAST1)* in tomato is specifically induced by GAs and is therefore used as a molecular marker to monitor changes in endogenous active GA levels.^39–41^ In transgenic lines L10 and L7, the transcript levels of *SlGAST1* were downregulated (Figure 6c). As expected, GA_3_ treatment induced *SlGAST1* expression, while PAC treatment reduced *SlGAST1* expression. These data provide additional evidence that fruit-specific overexpression of *SlGA2ox1* reduces the active GA concentrations in tomato fruits.

Because GAs promote cell elongation and expansion,^42^ we also analyzed the expression of cell expansion-related genes in tomato fruits. *SlEXP2*, *SlEXP8*, *SlEXP12* and *SlXTH9* were upregulated in GA_3_-treated fruits and significantly downregulated in PAC-treated tomato fruits in comparison with wild-type fruits (Figure 6d). The expression levels of these genes were decreased in transgenic fruits, which was consistent with the effects of PAC treatment.

## Discussion

Seven and eleven *GA2ox* genes have been reported in *Arabidopsis* and rice, respectively.^22^ Serrani *et al.*^6^ identified only five putative *GA2oxs* in tomato (*SlGA2ox1*- *SlGA2ox5*),^6^ however, in this study, eleven *GA2ox* genes were identified in the tomato genome, six of which (*SlGA2ox6*- *SlGA2ox11*) were newly identified (Supplementary Table S1). There are two possible explanations for this difference. One is the lack of sequence information available to Serrani *et al.* in 2007,^6^ because the tomato genome sequence only became available in 2012.^43^ The other possibility is that the identification of SlGA2oxs by Serrani *et al.* in 2007^6^ using the SlGA2ox1 amino-acid sequence as a query, may have resulted in a failure to identify other SlGA2ox proteins. This argument is supported by the fact that the method can identify some subgroups I and II GA2oxs, but not subgroup III GA2oxs.

Several GA2oxs from diverse plant species have been shown to possess the ability to deactivate C19 GAs or C20 GAs through enzyme assays.^11–14,16–19,21,44–48^ In tomato, SlGA2ox1, SlGA2ox3, SlGA2ox4 and SlGA2ox5 can deactivate C19 GAs, while SlGA2ox2 cannot deactivate C19 GAs or C20 GAs despite harboring all of the unique amino acids sequences conserved in GA2oxs.^6^ Serrani *et al.* speculated that this might be due to the replacement of a W (conserved in all GA2ox proteins of group I) by an R at position 92.^6^ On the basis of the motif analysis and MSAs of GA2ox proteins, we speculate that SlGA2ox6 and SlGA2ox11 have C19 GA2ox activity; SlGA2ox7, SlGA2ox8 and SlGA2ox9 have C20 GA2ox activity; and SlGA2ox10 is inactive against C20 GA2ox. Future studies are required to clarify this matter.

Globally or constitutively active gene promoters can be used to control the expression of GA biosynthesis or deactivation genes. However, due to global or constitutive alterations in GA contents in transgenic plants, it is difficult to distinguish the direct and indirect effects of GAs on fruit growth and development. For example, reductions in GA levels in leaf and stem tissues may influence photosynthetic activity, source–sink relations between leaves and fruits, plant stature, and can indirectly affect the growth and development of fruits.^28–30^ Therefore, fruit-specific overexpression of *GA2oxs* may provide an alternative method to study the role of GAs in fruit development because it does not affect the growth and development of vegetative organs, and reduces the endogenous active GA concentrations only in developing fruits.

In this study, the fruit-specific promoter TFM7 was used to control the expression of *SlGA2ox1.* There was no visible change in the growth and development of the vegetative organs of transgenic plants, but fruit weight were reduced. Although GA concentrations were not quantified, the reduction in fruit weight were likely due to reduced endogenous active GA concentrations, for the following reasons: (1) Serrani *et al.*^6^ reported that SlGA2ox1 metabolizes active GA_1_ and GA_4_ to inactive GA_8_ and GA_34_;^6^ (2) expression of the GA-response gene *SlGAST1* was downregulated in transgenic tomato; (3) the reduced fruit weight of transgenic tomato could be restored by exogenous applications of GA_3_; (4) treatment with GA biosynthesis inhibitor also reduced fruit weight that were similar to transgenic tomato; and (5) Martinez-Bello *et al.*^31^ reported that downregulating the expression of five GA2ox genes (*SlGA2ox1- SlGA2ox5*) in tomato resulted in a significant increase in active GA_4_ content and a reduction in inactive GA_34_ content.^31^

The effects of GAs on tomato fruit growth and development by means of chemical treatment, constitutive overexpression or silencing of GA biosynthetic and catabolic genes have been reported.^25–31^ Application of exogenous GA_3_ to unpollinated tomato ovaries or tomatoes overexpressing *GA20ox1* results in an increased fruit set, but the fruits are parthenocarpic, with reduced size and weight compared with pollinated wild-type fruits.^25,28^ In contrast, Serrani *et al.*^6^ applied two different inhibitors of GA biosynthesis (LAB 198999 and paclobutrazol) to pollinated ovaries, which resulted in a decreased fruit set and size compared with untreated pollinated fruits.^6^ Xiao *et al.*^30^ and Olimpieri *et al.*^29^ reported that constitutive co-suppression of G*A20oxs* in tomato leads to reduced GA concentrations and severe defects in vegetative and reproductive development,^29,30^ but they did not report fruit size and weight. In our study, with transgenic manipulation of GA contents in fruit tissues specific, we observed that overexpression of *SlGA2ox1* led to reductions in fruit weight, which was similar to the effects of GA biosynthesis inhibitors reported by Serrani *et al.*^6^ In addition, Serrani *et al.*^42^ demonstrated that GA_3_ treatment results in larger cells compared with untreated fruits,^42^ in this study, the expressions of some cell expansion-related genes was decreased in transgenic fruits. Thus, we suggest that overexpression of *SlGA2ox1* in tomato fruits reduced the active GA contents, and downregulated the expression of cell expansion genes. This led to small cells and reduced fruit growth, as evidenced by reduced fruit weight.

Analysis of GA-deficient mutants has provided evidence that GAs are necessary for seed development and germination in *Arabidopsis*, tomato and pea.^5,49,50^ Swain *et al.* proposed that this may be due to poor utilization of assimilates or disruption to the relative growth rates of the embryo and endosperm.^49^ Using a transgenic method, Singh *et al.*^51^ reported that constitutive overexpression of pea *GA2ox2* caused seed abortion in *Arabidopsis*,^51^ Moreover, Singh *et al.*^52^ used the MEDEA promoter to control expression of the pea *GA2ox2* in young seeds and vegetative tissues of *Arabidopsis*, seeds were aborted, and the ovule numbers and silique length were reduced.^52^ These results suggest that GAs play an essential role in seed development. In this study, tomato fruit-specific overexpression of *SlGA2ox1* resulted in reduced seed number and germination, suggesting that overexpression of *SlGA2ox1* in tomato fruit reduced the active GA contents, which suppressed seed development and germination.

Santino *et al.*^37^ reported that the fruit-specific promoter TFM7 is mainly active in the pericarp, columella and placental tissues of immature green fruits of tomato.^37^ Our data suggest that the GA contents of the sarcocarp were reduced by fruit-specific overexpression of *SlGA2ox1*. However, Santino *et al.*^37^ did not determine whether the TFM7 promoter is active in seeds,^37^ and therefore the effects on seed GA contents are unclear. If the TFM7 promoter is not active in seeds, the inhibition of seed growth and development may be caused by reduced GA transport from the sarcocarp. However, if the TFM7 promoter is active in seeds, fruit-specific overexpression of *SlGA2ox1* would reduce the GA content of both the sarcocarp and seeds, leading to inhibition of seed growth and development.

In this study, 11 GA2oxs were identified in tomato. Motif analysis and MSAs suggested that SlGA2ox1 to SlGA2ox6 and SlGA2ox11 are C19 GA2oxs, while SlGA2ox7 to SlGA2ox10 are C20 GA2oxs. The findings suggested that *SlGA2ox1* is responsible for the deactivation of active GAs in tomato plants. Moreover, GAs likely play an important role in tomato fruit and seed growth and development, including being a determinant of seed germination rate.

## References

[bib1] Ranjan A, Ichihashi Y, Sinha NR. The tomato genome: implications for plant breeding, genomics and evolution. Genome Biol 2012; 13: 167–174. 22943138 10.1186/gb-2012-13-8-167 PMC3491363

[bib2] Srivastava A, Handa AK. Hormonal regulation of tomato fruit development: a molecular perspective. J Plant Growth Regul 2005; 24: 67–82.

[bib3] Ozga JA, Reinecke DM. Hormonal interactions in fruit development. J Plant Growth Regul 2003; 22: 73–81.

[bib4] Seymour GB, Østergaard L, Chapman NH, Knapp S, Martin C. Fruit development and ripening. Annu Rev Plant Biol 2013; 64: 219–224. 23394500 10.1146/annurev-arplant-050312-120057

[bib5] Groot SPC, Bruinsma J, Karssen CM. The role of endogenous gibberellin in seed and fruit development of tomato: studies with a gibberellin-deficient mutant. Physiol Plant 1987; 71: 184–190.

[bib6] Serrani JC, Sanjuán R, Ruiz-Rivero O, Fos M, García-Martínez JL. Gibberellin regulation of fruit set and growth in tomato. Plant Physiol 2007; 145: 246–257. 17660355 10.1104/pp.107.098335 PMC1976567

[bib7] de Jong M, Mariani C, Vriezen WH. The role of auxin and gibberellin in tomato fruit set. J Exp Bot 2009; 60: 1523–1532. 19321650 10.1093/jxb/erp094

[bib8] Hedden P, Thomas SG. Gibberellin biosynthesis and its regulation. Biochem J 2012; 444: 11–25. 22533671 10.1042/BJ20120245

[bib9] Yamaguchi S. Gibberellin metabolism and its regulation. Annu Rev Plant Biol 2008; 59: 225–251. 18173378 10.1146/annurev.arplant.59.032607.092804

[bib10] Olszewski N, Sun TP, Gubler F. Gibberellin signaling biosynthesis, catabolism, and response pathways. Plant Cell 2002; 14: S61–S80. 12045270 10.1105/tpc.010476 PMC151248

[bib11] Thomas SG, Phillips AL, Hedden P. Molecular cloning and functional expression of gibberellin 2-oxidases, multifunctional enzymes involved in gibberellin deactivation. Proc Natl Acad Sci USA 1999; 96: 4698–4703. 10200325 10.1073/pnas.96.8.4698 PMC16395

[bib12] Yamauchi Y, Takeda-Kamiya N, Hanada A, Ogawa M, Kuwahara A, Seo M et al. Contribution of gibberellin deactivation by AtGA2ox2 to the suppression of germination of dark-imbibed *Arabidopsis thaliana* seeds. Plant Cell Physiol 2007; 48: 555–556. 17289793 10.1093/pcp/pcm023

[bib13] Wang H, Caruso LV, Downie AB, Perry SE. The embryo MADS domain protein AGAMOUS-Like 15 directly regulates expression of a gene encoding an enzyme involved in gibberellin metabolism. Plant Cell 2004; 16: 1206–1219. 15084721 10.1105/tpc.021261 PMC423210

[bib14] Sakai M, Sakamoto T, Saito T, Perry SE. Expression of novel rice gibberellin 2-oxidase gene is under homeostatic regulation by biologically active gibberellins. J Plant Res 2003; 116: 161–164. 12736788 10.1007/s10265-003-0080-z

[bib15] Shan C, Mei Z, Duan J, Chen H, Feng H, Cai W. OsGA2ox5, a gibberellin metabolism enzyme, is involved in plant growth, the root gravity response and salt stress. PLoS One 2014; 9: e87110. 24475234 10.1371/journal.pone.0087110 PMC3903634

[bib16] Huang J, Tang D, Shen Y, Qin B, Hong L, You A et al. Activation of gibberellin 2-oxidase 6 decreases active gibberellin levels and creates a dominant semi-dwarf phenotype in rice (*Oryza sativa* L.). J Genet Genomics 2010; 37: 23–36. 20171575 10.1016/S1673-8527(09)60022-9

[bib17] Lee DH, Lee IC, Kim KJ, Kim DS, Na HJ, Lee IJ et al. Expression of *gibberellin 2-oxidase 4* from *Arabidopsis* under the control of a senescence-associated promoter results in a dominant semi-dwarf plant with normal flowering. J Plant Biol 2014; 57: 106–116.

[bib18] Schomburg FM, Bizzell CM, Lee DJ, Zeevaart JA, Amasino RM. Overexpression of a novel class of gibberellin 2-oxidases decreases gibberellin levels and creates dwarf plants. Plant Cell 2003; 15: 151–163. 12509528 10.1105/tpc.005975 PMC143488

[bib19] Sakamoto T, Kobayashi M, Itoh H, Tagiri A, Kayano T, Tanaka H et al. Expression of a gibberellin 2-oxidase gene around the shoot apex is related to phase transition in rice. Plant Physiol 2001; 125: 1508–1516. 11244129 10.1104/pp.125.3.1508 PMC65628

[bib20] Sakamoto T, Morinaka Y, Ishiyama K, Kobayashi M, Itoh H, Kayano T et al. Genetic manipulation of gibberellin metabolism in transgenic rice. Nat Biotechnol 2003; 21: 909–913. 12858182 10.1038/nbt847

[bib21] Lo SF, Yang SY, Chen KT, Hsing YI, Zeevaart JA, Chen LJ et al. A novel class of gibberellin 2-oxidases control semidwarfism, tillering, and root development in rice. Plant Cell 2008; 20: 2603–2618. 18952778 10.1105/tpc.108.060913 PMC2590730

[bib22] Wuddineh WA, Mazarei M, Zhang J, Poovaiah CR, Mann DG, Ziebell A et al. Identification and overexpression of *gibberellin 2-oxidase* (*GA2ox*) in switchgrass (*Panicum virgatum* L.) for improved plant architecture and reduced biomass recalcitrance. Plant Biotechnol J 2015; 13: 636–647. 25400275 10.1111/pbi.12287

[bib23] Agharkar M, Lomba P, Altpeter F, Zhang H, Kenworthy K, Lange T. Stable expression of *AtGA2ox1* in a low-input turfgrass (*Paspalum notatum* Flugge) reduces bioactive gibberellin levels and improves turf quality under field conditions. Plant Biotechnol J 2007; 5: 791–801. 17764521 10.1111/j.1467-7652.2007.00284.x

[bib24] Zhao XY, Zhu DF, Zhou B, Peng WS, Lin JZ, Huang XQ et al. Over-expression of the *AtGA2ox8* gene decreases the biomass accumulation and lignification in rapeseed (*Brassica napus* L.). J Zhejiang Univ Sci B 2010; 11: 471–481. 20593511 10.1631/jzus.B1000161 PMC2897016

[bib25] Bünger-Kibler S, Bangerth F. Relationship between cell number, cell size and fruit size of seeded fruits of tomato (*Lycopersicon esculentum* Mill.), and those induced parthenocarpically by the application of plant growth regulators. Plant Growth Regul 1982; 1: 143–154.

[bib26] Fos M, Nuez F, Garcìa-Martìnez JL. The gene *pat-2*, which induces natural parthenocarpy, alters the gibberellin content in unpollinated tomato ovaries. Plant Physiol 2000; 122: 471–480. 10677440 10.1104/pp.122.2.471 PMC58884

[bib27] Olimpieri I, Siligato F, Caccia R, Mariotti L, Ceccarelli N, Soressi GP et al. Tomato fruit set driven by pollination or by the parthenocarpic fruit allele are mediated by transcriptionally regulated gibberellin biosynthesis. Planta 2007; 226: 877–888. 17503074 10.1007/s00425-007-0533-z

[bib28] García-Hurtado N, Carrera E, Ruiz-Rivero O, Hedden P, Gong F, García-Martínez JL. The characterization of transgenic tomato overexpressing gibberellin 20-oxidase reveals induction of parthenocarpic fruit growth, higher yield, and alteration of the gibberellin biosynthetic pathway. J Exp Bot 2012; 63: 5803–5813. 22945942 10.1093/jxb/ers229

[bib29] Olimpieri I, Caccia R, Picarella ME, Pucci A, Santangelo E, Soressi GP et al. Constitutive co-suppression of the *GA 20-oxidase 1* gene in tomato leads to severe defects in vegetative and reproductive development. Plant Sci 2011; 180: 496–503. 21421397 10.1016/j.plantsci.2010.11.004

[bib30] Xiao J, Li H, Zhang J, Chen R, Zhang Y, Ouyang B et al. Dissection of GA 20-oxidase members affecting tomato morphology by RNAi-mediated silencing. Plant Growth Regul 2006; 50: 179–189.

[bib31] Martínez-Bello L, Moritz T, López-Díaz I. Silencing C19-GA 2-oxidases induces parthenocarpic development and inhibits lateral branching in tomato plants. J Exp Bot 2015; 66: 5897–5910. 26093022 10.1093/jxb/erv300 PMC4566981

[bib32] Finn RD, Coggill P, Eberhardt RY, Eddy SR, Mistry J, Mitchell AL et al. The Pfam protein families database: towards a more sustainable future. Nucleic Acids Res 2016; 44: D279–D285. 26673716 10.1093/nar/gkv1344 PMC4702930

[bib33] Eddy SR. Profile hidden Markov models. Bioinformatics 1998; 14: 755–763. 9918945 10.1093/bioinformatics/14.9.755

[bib34] Bailey TL, Boden M, Buske FA, Frith M, Grant CE, Clementi L et al. MEME SUITE: tools for motif discovery and searching. Nucleic Acids Res 2009; 37: W202–W208. 19458158 10.1093/nar/gkp335 PMC2703892

[bib35] Larkin MA, Blackshields G, Brown NP, Chenna R, McGettigan PA, McWilliam H et al. Clustal W and Clustal X version 2.0. Bioinformatics 2007; 23: 2947–2948. 17846036 10.1093/bioinformatics/btm404

[bib36] Fillatti JJ, Kiser J, Rose R, Comai L. Efficient transfer of a glyphosate tolerance gene into tomato using a binary *Agrobacterium tumefaciens* vector. Nat Biotechnol 1987; 5: 726–730.

[bib37] Santino CG, Stanford GL, Conner TW. Developmental and transgenic analysis of two tomato fruit enhanced genes. Plant Mol Biol 1997; 33: 405–416. 9049262 10.1023/a:1005738910743

[bib38] Davuluri GR, van Tuinen A, Fraser PD, Manfredonia A, Newman R, Burgess D et al. Fruit-specific RNAi-mediated suppression of *DET1* enhances carotenoid and flavonoid content in tomatoes. Nat Biotechnol 2005; 23: 890–895. 15951803 10.1038/nbt1108 PMC3855302

[bib39] Ding J, Chen B, Xia X, Mao W, Shi K, Zhou Y et al. Cytokinin-induced parthenocarpic fruit development in tomato is partly dependent on enhanced gibberellin and auxin biosynthesis. PLoS One 2013; 8: e70080. 23922914 10.1371/journal.pone.0070080 PMC3726760

[bib40] Serrani JC, Ruiz-Rivero O, Fos M, García-Martínez JL. Auxin-induced fruit-set in tomato is mediated in part by gibberellins. Plant J 2008; 56: 922–934. 18702668 10.1111/j.1365-313X.2008.03654.x

[bib41] Shi L, Olszewski NE. Gibberellin and abscisic acid regulate *GAST1* expression at the level of transcription. Plant Mol Biol 1998; 38: 1053–1060. 9869411 10.1023/a:1006007315718

[bib42] Serrani JC, Fos M, Atarés A, García-Martínez JL. Effect of gibberellin and auxin on parthenocarpic fruit growth induction in the cv Micro-Tom of tomato. J Plant Growth Regul 2007; 26: 211–221.

[bib43] Tomato Genome Consortium. The tomato genome sequence provides insights into fleshy fruit evolution. Nature 2012; 485: 635–641. 22660326 10.1038/nature11119 PMC3378239

[bib44] Lee DJ, Zeevaart JA. Molecular cloning of *GA 2-oxidase3* from spinach and its ectopic expression in *Nicotiana sylvestris*. Plant Physiol 2005; 138: 243–254. 15821147 10.1104/pp.104.056499 PMC1104179

[bib45] Martin DN, Proebsting WM, Hedden P. The *SLENDER* gene of pea encodes a gibberellin 2-oxidase. Plant Physiol 1999; 121: 775–781. 10557225 10.1104/pp.121.3.775 PMC59439

[bib46] Lester DR, Ross JJ, Smith JJ, Elliott RC, Reid JB. Gibberellin 2-oxidation and the *SLN* gene of *Pisum sativum*. Plant J 1999; 19: 65–73. 10417727 10.1046/j.1365-313x.1999.00501.x

[bib47] Lee Y, Kim Y, Kim SY, Lee IJ, Choi D, Paek KH et al. A novel gibberellin 2-oxidase gene *CaGA2ox1* in pepper is specifically induced by incompatible plant pathogens. Plant Biotechnol Rep 2012; 6: 381–390.

[bib48] Nakaminami K, Sawada Y, Suzuki M, Kenmoku H, Kawaide H, Mitsuhashi W et al. Deactivation of gibberellin by 2-oxidation during germination of photoblastic lettuce seeds. Biosci Biotechnol Biochem 2003; 67: 1551–1558. 12913300 10.1271/bbb.67.1551

[bib49] Swain SM, Reid JB, Kamiya Y. Gibberellins are required for embryo growth and seed development in pea. Plant J 1997; 12: 1329–1338.

[bib50] Barendse GWM, Kepczynski J, Karssen CM, Koornneef M. The role of endogenous gibberellins during fruit and seed development: studies on gibberellin-deficient genotypes of *Arabidopsis thaliana*. Physiol Plantarum 1986; 67: 315–319.

[bib51] Singh DP, Jermakow AM, Swain SM. Gibberellins are required for seed development and pollen tube growth in *Arabidopsis*. Plant Cell 2002; 14: 3133–3147. 12468732 10.1105/tpc.003046 PMC151207

[bib52] Singh DP, Filardo FF, Storey R, Jermakow AM, Yamaguchi S, Swain SM. Overexpression of a gibberellin inactivation gene alters seed development, *KNOX* gene expression, and plant development in *Arabidopsis*. Physiol Plant 2010; 138: 74–90. 19825007 10.1111/j.1399-3054.2009.01289.x

