# Supplementary figures and images for: Identification and characterization of tomato gibberellin 2-oxidases (GA2oxs) and effects of fruit-specific SlGA2ox1 overexpression on fruit and seed growth and development
Source: Hortic Res. 2016 Dec 7;3:16059–. doi: 10.1038/hortres.2016.59 (PMC5142509; doi:10.1038/hortres.2016.59)

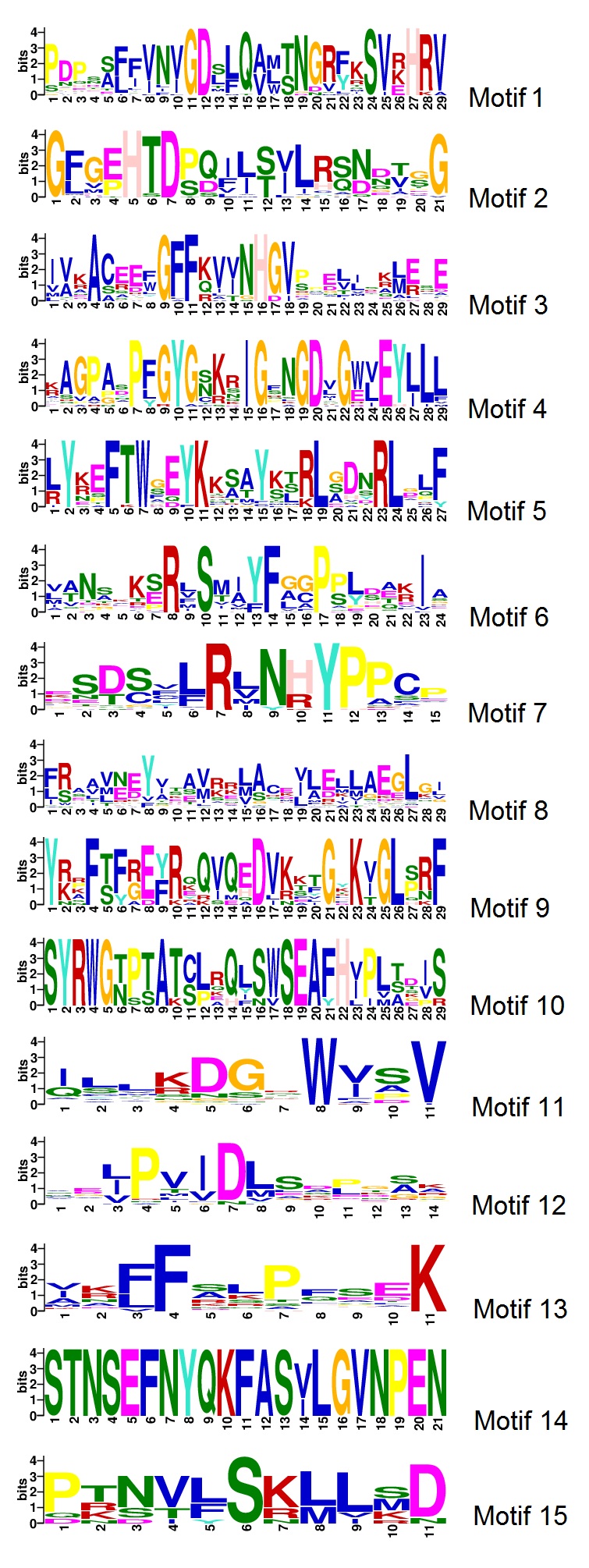

Supplement: Supplementary Figure S1 [file hortres201659-s1.jpg]

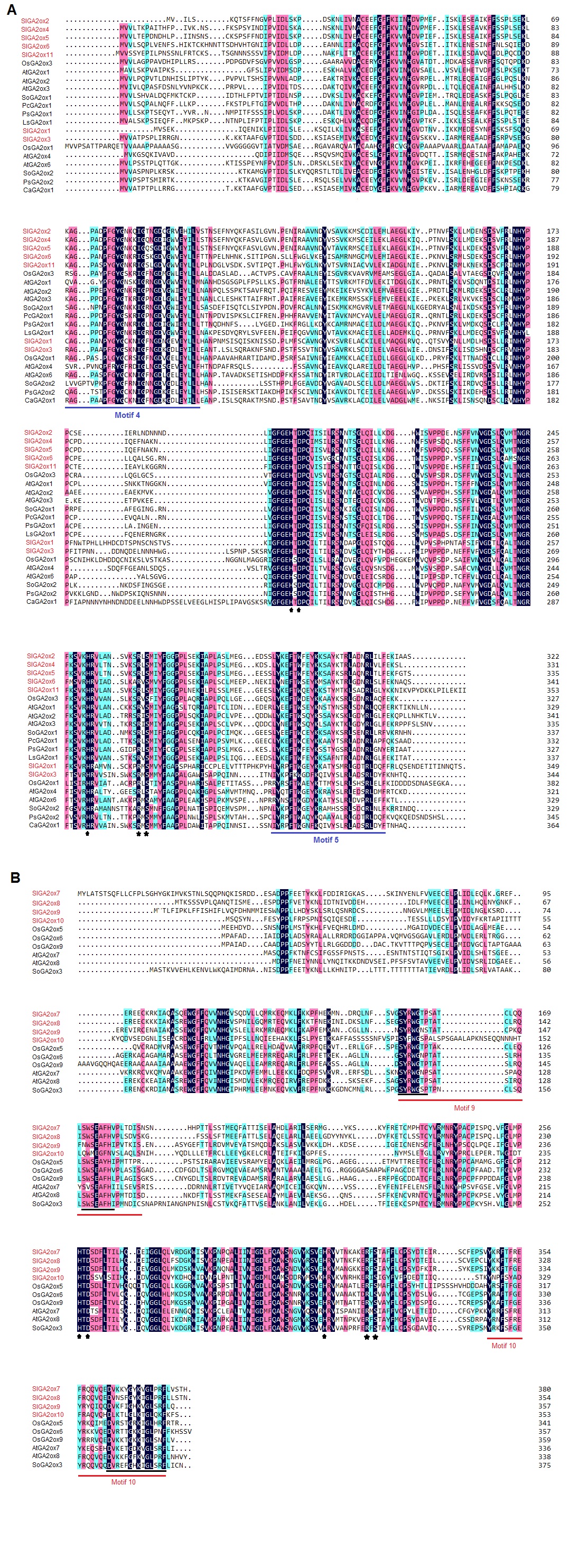

Supplement: Supplementary Figure S2 [file hortres201659-s2.jpg]

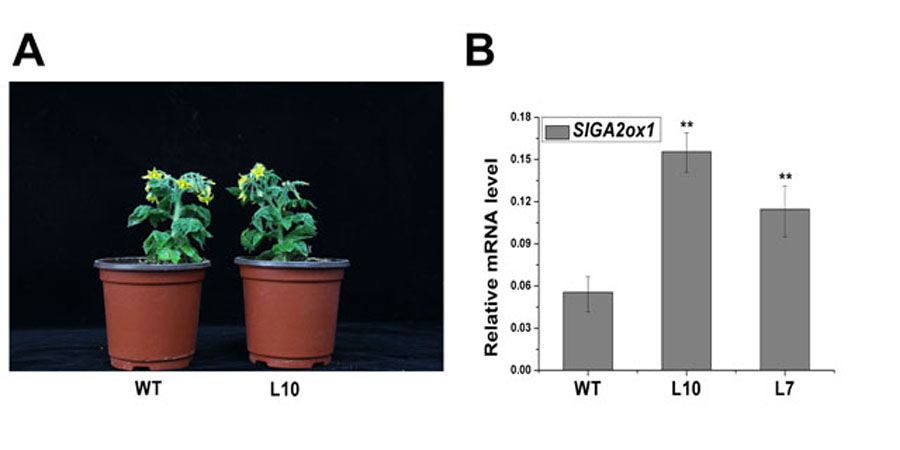

Supplement: Supplementary Figure S3 [file hortres201659-s3.jpg]
