# Supplementary material for: Identification and characterization of tomato gibberellin 2-oxidases (GA2oxs) and effects of fruit-specific SlGA2ox1 overexpression on fruit and seed growth and development
Source: Hortic Res. 2016 Dec 7;3:16059–. doi: 10.1038/hortres.2016.59 (PMC5142509; doi:10.1038/hortres.2016.59)
Supplement: Supplementary Tables [file hortres201659-s4.doc]

Supplemental Table S1. Sequences of the primer pairs for RT-qPCR

| Gene name | The forward primer sequence (5´-3´) | The reverse primer sequence (5´-3´) |
| --- | --- | --- |
| *SlGA20ox2* | 5´GTGATCCGATTGCAGCTAAGCG 3´ | 5´AGCGATGTGCAAGTGAGATAAG 3´ |
| *SlGA20ox3* | 5´CTAGTGTTACTAGAGAACTACA 3´ | 5´TGTCAACCCCATGGTTAACCAC 3´ |
| *SlGA3ox1* | 5´GAATCCCATGCATGGACATCAT 3´ | 5´TGTTATCGAGGTCGATCACTGG 3´ |
| *SlGA3ox2* | 5´CATTGGACGACGATGGATCGCG 3´ | 5´GCATGCATGGCCAATTGTATCC 3´ |
| *SlGA2ox1* | 5´CATAGTGAAAGCCTCTGAAG 3´ | 5´CAACTTCACCATTATCTCCA 3´ |
| *SlGA2ox2* | 5´CTCATCGTTAATGCCTGCGAAG 3´ | 5´ACTTGATGGCTTCGGATTCGAG 3´ |
| *SlGA2ox3* | 5´GATGACAAGTCTATTGCGTCCG 3´ | 5´CAGCGCGTCCCTTCTCAGAGAC3´ |
| *SlGA2ox4* | 5´CTCATCGTTAATGCCTGCGAAG 3´ | 5´GATCAGCAGGCCCTGCCTTTAG 3´ |
| *SlGA2ox5* | 5´GAACCTCATCGTTGAGGCCTGC 3´ | 5´GATGGCTTCGGATTCGAGTTTAC 3´ |
| *SlGA2ox6* | 5´GTGATCATGTTCACACTGGT 3´ | 5´GGACAAGTTGAAGAAGTTGA 3´ |
| *SlGA2ox7* | 5´GGTGAAGTCTACGAACTTGTC 3´ | 5´CAATTCACATTCTTCAACCAC 3´ |
| *SlGA2ox8* | 5´CACCTGATCCTCCATTTGTGG 3´ | 5´GCGATGCCTTGGCTATCTTC 3´ |
| *SlGA2ox9* | 5´GATAGAGTCTTGGAATCCTCC 3´ | 5´CATCGAATCACTTCTCTCTCG 3´ |
| *SlGA2ox10* | 5´CGACCATCGCCAAACATCTCT 3´ | 5´CAATCTCTACAAGCTTCACTG 3´ |
| *SlGA2ox11* | 5´CATGCAAATCTACCTCTGGT 3´ | 5´ACTGTACTGCATCACTTTCC 3´ |
| *SlEXP2* | 5´CTGATTATGGAGGATGGCAAAC 3´ | 5´GTACTTAGTGCTGCAGTGTTAG 3´ |
| *SlEXP8* | 5´CTCAGCAGATTCTGGATGGA 3´ | 5´CATTGAACAATGCTGTACTC 3´ |
| *SlEXP12* | 5´GGTGATGCTTCTGGAACAATGG 3´ | 5´AGAACCACAGCTCAACCCATTG 3´ |
| *SlGAST1* | 5´GCACGTACCGGTGTTCAAAGAC 3´ | 5´CAGTTATTGTAGCAAGGGCAAC3 ´ |
| *SlXTH9* | 5´GTATCCAATTACTAGCCTCAG 3´ | 5´GTTGTTACAGTGCCAGCAGAG 3´ |
| *SlActin* | 5´CATAGTGAAAGCCTCTGAAG 3´ | 5´GAGCTTCATCACCCACATAC 3´ |

Supplemental Table S2. Detailed information of the GA2ox proteins from tomato

| **Class** | **Gene Name** | **Sequence ID** | **Chr** | **Protein Length (aa)** | **pI** | **M (Kda)** |
| --- | --- | --- | --- | --- | --- | --- |
| Ⅰ | SlGA2ox2 | NP_001234338 | 7 | 322 | 5.99 | 36.05 |
|  | SlGA2ox4 | NP_001234752 | 7 | 331 | 9.17 | 37.21 |
|  | SlGA2ox5 | NP_001234757 | 7 | 335 | 6.52 | 37.59 |
|  | SlGA2ox6 | XP_004233129 | 2 | 341 | 6.78 | 38.17 |
|  | SlGA2ox11 | XP_010324754 | 8 | 354 | 8.10 | 39.68 |
| II | SlGA2ox1 | NP_001234865 | 5 | 349 | 7.98 | 39.34 |
|  | SlGA2ox3 | NP_001234747 | 1 | 344 | 7.67 | 38.54 |
| Ⅲ | SlGA2ox7 | XP_004232746 | 2 | 380 | 8.15 | 44.12 |
|  | SlGA2ox8 | XP_004248394 | 10 | 354 | 5.15 | 40.84 |
|  | SlGA2ox9 | XP_004237179 | 4 | 357 | 7.54 | 41.42 |
|  | SlGA2ox10 | XP_004242519 | 6 | 353 | 5.80 | 40.57 |

Supplemental Table S3. The identical and similarity percentage between the different SlGA2ox proteins

| Protein | SlGA2ox1 | SlGA2ox2 | SlGA2ox3 | SlGA2ox4 | SlGA2ox5 | SlGA2ox6 | SlGA2ox7 | SlGA2ox8 | SlGA2ox9 | SlGA2ox10 |
| --- | --- | --- | --- | --- | --- | --- | --- | --- | --- | --- |
| SlGA2ox2 | 46.44 |  |  |  |  |  |  |  |  |  |
| SlGA2ox3 | 54.46 | 50.76 |  |  |  |  |  |  |  |  |
| SlGA2ox4 | 48.13 | 85.94 | 47.63 |  |  |  |  |  |  |  |
| SlGA2ox5 | 45.31 | 84.86 | 46.59 | 89.12 |  |  |  |  |  |  |
| SlGA2ox6 | 45.90 | 63.14 | 44.00 | 59.00 | 60.65 |  |  |  |  |  |
| SlGA2ox7 | 28.35 | 27.39 | 28.30 | 26.58 | 27.30 | 28.33 |  |  |  |  |
| SlGA2ox8 | 29.18 | 26.25 | 26.86 | 26.75 | 26.14 | 28.51 | 68.50 |  |  |  |
| SlGA2ox9 | 28.53 | 30.33 | 28.39 | 29.87 | 30.43 | 29.36 | 55.33 | 48.35 |  |  |
| SlGA2ox10 | 25.00 | 29.89 | 26.99 | 26.41 | 27.68 | 26.65 | 36.61 | 33.63 | 33.61 |  |
| SlGA2ox11 | 46.37 | 61.97 | 41.87 | 62.54 | 60.82 | 63.64 | 26.26 | 25.69 | 27.60 | 30.16 |
